# Monthly Summary

**Published:** 1881-04

**Authors:** 


### MONTHLY SUMMARY.

*Bleaching Gutta Percha.*—Dissolve the gutta percha in twenty times its weight of boiling benzole, add to the solution plaster of very good quality, and agitate the mixture from time to time. By reposing for two days the plaster is deposited and carries down with it all the impurities of the gutta percha insoluble in benzole. The clear liquid decanted is introduced by small portions at a time into twice its volume of alcohol 90 per cent., agitating continually. During this operation the gutta percha is precipitated in the state of a pasty mass, perfectly white. The nesiccation of the gutta percha, thus purified, requires several weeks' exposure to the air, but may be accelerated by trituration in a mortar, which liberate moistures which it tends to retain.—*Druggists Circular.*

